# Anti-Müllerian hormone in African-American women with systemic lupus erythematosus

**DOI:** 10.1136/lupus-2020-000439

**Published:** 2020-11-01

**Authors:** Meghan Angley, Jessica B Spencer, S Sam Lim, Penelope P Howards

**Affiliations:** 1 Department of Epidemiology, Rollins School of Public Health, Emory University, Atlanta, Georgia, USA; 2 Division of Reproductive Endocrinology and Infertility, Department of Gynecology and Obstetrics, Emory University School of Medicine, Atlanta, Georgia, USA; 3 Department of Medicine, Division of Rheumatology, Emory University School of Medicine, Atlanta, Georgia, USA

**Keywords:** lupus erythematosus, systemic, cyclophosphamide, inflammation

## Abstract

**Objective:**

Women with SLE may experience ovarian insufficiency or dysfunction due to treatment or disease effects. Anti-Müllerian hormone (AMH), a marker of ovarian reserve, has been examined in small populations of women with SLE with conflicting results. To date, these studies have included very few African-American women, the racial/ethnic group at greatest risk of SLE.

**Methods:**

We enrolled African-American women aged 22–40 years diagnosed with SLE after age 17 from the Atlanta Metropolitan area. Women without SLE from the same area were recruited from a marketing list for comparison. AMH was measured in serum using the Ansh Labs assay (Webster, Texas, USA). We considered AMH levels <1.0 ng/mL and AMH <25th percentile of comparison women as separate dichotomous outcomes. Log-binomial regression models estimating prevalence ratios were adjusted for age, body mass index and hormonal contraception use in the previous year.

**Results:**

Our sample included 83 comparison women without SLE, 68 women with SLE and no history of cyclophosphamide (SLE/CYC−) and 11 women with SLE and a history of cyclophosphamide treatment (SLE/CYC+). SLE/CYC+ women had a greater prevalence of AMH <1.0 ng/mL compared with women without SLE (prevalence ratio (PR): 2.90, 95% CI: 1.29 to 6.51). SLE/CYC− women were also slightly more likely to have AMH <1.0 ng/mL (PR: 1.62, 95% CI: 0.93 to 2.82) than comparison women. Results were similar when considering AMH <25th percentile by age of comparison women.

**Conclusions:**

Treatment with CYC is associated with low AMH in African-American women with SLE. SLE itself may also be associated with reduced AMH, but to a lesser extent.

## Introduction

SLE is an autoimmune disease affecting multiple organ systems. SLE is nine times more common among women, and it is primarily diagnosed during women’s reproductive years.^1^ SLE is characterised by periods of quiescence and flares where the disease may be highly active.^2^ It is also associated with the potential for progressive, irreversible organ damage.^3^ Over half of patients with SLE show at least some organ damage after 5 years of the disease.^4^ Commonly affected organs include the skin, musculoskeletal system, and heart. In women, organ damage can also manifest as ovarian insufficiency or dysfunction.

Anti-Müllerian hormone (AMH) is a marker of ovarian reserve. Unlike follicle-stimulating hormone, AMH levels remain relatively constant throughout a woman’s menstrual cycle so they can be measured at any point during the menstrual cycle. AMH is a hormone secreted by granulosa cells of small antral follicles and correlates with the number of developing follicles in the ovaries. It is a marker of ovarian reserve and therefore declines throughout a woman’s reproductive lifespan.^5^ AMH has clinical utility as an indicator of a woman’s response to ovarian stimulation for in vitro fertilisation.^6^ Several studies have also found AMH to be somewhat predictive of time-to-menopause.^7–9^

AMH has been examined in women with SLE in several studies, but with conflicting results. Generally, there is a consensus that AMH is lower among women with SLE treated with cyclophosphamide (CYC).^10–13^ However, while some found a substantial difference in AMH levels between women with SLE not treated with CYC and women without SLE,^14–16^ others did not.^10 17^ Overall, these studies have varying limitations. Most include fewer than 50 women with SLE and some lack comparison groups of women without SLE. The largest study included 112 women with SLE, but did not include a comparison group of women without SLE and instead compared women who had been treated with CYC with women with SLE who had never been treated with CYC.^12^ One study did not distinguish between women with SLE who had been treated with CYC and those that had not.^18^ In addition, while 10% of the study population in the study by Marder *et al* was classified as ‘non-white’, no studies specifically examine AMH among African-American or black women with SLE.^13^ In the USA, African-American women are at 3 times the risk of SLE compared with white women and also tend to experience more severe SLE, potentially causing greater ovarian toxicity.^19–21^ Thus, the relationship between SLE and ovarian reserve as measured by AMH may differ among African-American women.

In this analysis, we examined AMH levels in a sample of African-American women with SLE, compared with African-American women without SLE. We distinguished between women with SLE who had a history of CYC treatment and women with SLE who had never been treated with CYC. We hypothesised that women with SLE would have lower AMH than women without SLE after controlling for age, and that women with a history of treatment with CYC would have the lowest AMH levels.

## Methods

### Study participants

Women with SLE were enrolled from the Georgians Organized Against Lupus (GOAL) Cohort, an ongoing cohort of patients with validated cases of SLE from the Atlanta metropolitan area. The primary source of recruited participants for GOAL was the Georgia Lupus Registry (GLR), a population-based registry of all SLE cases in Fulton and DeKalb counties in Georgia.^20^ Additional patients in GOAL were enrolled from Emory Healthcare, Grady Memorial Hospital and diverse community rheumatology clinics. The case definition of SLE was meeting at least four of the American College of Rheumatology (ACR) Classification criteria for SLE^22^ (GLR, GOAL) or meeting three ACR criteria with a final diagnosis of SLE by a board-certified rheumatologist (GLR).

One hundred women were recruited from GOAL for the Lupus Impacting the Female Experience (LIFE) Study, a pilot study examining women’s reproductive and fertility histories while living with SLE. Women were recruited by phone and email notices and in person at lupus clinics at Emory Healthcare and Grady Memorial Hospital. Eligible women were aged 22–40 years at the time of enrolment and had been diagnosed with SLE at age 18–35 years. Since part of the study objectives were to examine women’s reproductive goals, we restricted enrolment to women who were more likely to be potentially planning pregnancies and also only included women who had been diagnosed with SLE as adults as treatment regimens differ for those diagnosed with SLE as children. We also restricted our sample to women who had never had a hysterectomy or a diagnosis of cancer and were not currently receiving kidney dialysis. Women were interviewed in-person about their medical and reproductive histories, and at the time of interview, their height and weight were measured and a blood sample was taken by a trained research phlebotomist. Race was self-identified in the study interview. All participants were compensated for their time and travel. Participants provided written informed consent. This analysis was restricted to the African-American participants in the pilot study (89% of participants).

Data on comparison women were obtained from the Furthering Understanding of Cancer, Health and Survivorship in Adult (FUCHSIA) Women’s Study.^23^ Comparison women in the FUCHSIA Women’s Study were originally recruited from marketing lists to serve as the general population comparison for female cancer survivors. We restricted the FUCHSIA Women’s Study comparison group to women 22–40 years of age, who had never had a hysterectomy, and who lived in the metropolitan Atlanta area at the time of the interview to make them comparable to the women with SLE enrolled in the LIFE study. The comparison women completed an interview similar to the one used in the LIFE study and provided oral consent at the time of interview. A subset of the comparison women in FUCHSIA completed clinic visits where they provided blood samples and their height and weight were measured by study staff.

Among both the SLE and the comparison women, we excluded those with a self-reported history of polycystic ovarian syndrome (PCOS). Women with PCOS typically have very high AMH levels which may skew interpretation for the rest of the sample.^5^ We defined hormonal contraception use in the past year as use of oral contraceptive pills, the patch, the NuvaRing, Depo-Provera or the subdermal implant in the previous 12 months, but excluded the hormonal intrauterine device. We created three exposure categories: women with SLE with no history of CYC treatment (SLE/CYC−), women with SLE who had been treated with CYC (SLE/CYC+) and comparison women.

### Patient and public involvement

Patients were not involved in the design or analysis of this study.

### AMH assay

Serum AMH was measured among women in LIFE and FUCHSIA using the same ELISA (UltraSensitive AMH/MIS ELISA, Ansh Labs, Webster, Texas, USA). Age at interview is the same age at which AMH was measured. Serum AMH was measured in duplicate and samples were processed by the University of Southern California Reproductive Endocrinology Laboratory. The limit of detection (LOD) of the assay was 0.076 ng/mL. For participants whose values were found to be below the LOD, they were assigned a value of $\frac{LOD}{\sqrt{2}}$ (0.054 ng/mL). In FUCHSIA, an additional assay with greater sensitivity was conducted for participants whose AMH levels were undetectable by the UltraSensitive assay. To facilitate comparisons between the participants in LIFE and FUCHSIA, we set the AMH values for all FUCHSIA participants who were assessed with the more sensitive assay to $\frac{LOD}{\sqrt{2}}$ of the UltraSensitive assay (0.054 ng/mL).

### Statistical analysis

Participant characteristics were determined from data collected during interviews. Height and weight measurements were used to calculate participant body mass index (BMI) in kg/m^2^. Menopause was defined as amenorrhoea lasting 12 months or more without the resumption of menses while not using hormonal contraception or pregnant, and with AMH levels below the LOD.

We examined ‘low AMH’ using two different definitions: AMH <1.0 ng/mL at any age and AMH <25th age group-specific percentile of women without SLE. In a literature review to find appropriate AMH standards by age, we found several age-specific population standards.^24–28^ However, these population standards varied widely from each other, and none were good substitutes for our source population: African-American women in the Atlanta metropolitan area. Therefore, we generated our own AMH standards by age using the comparison population of African-American women without SLE. Using our comparison women without SLE as the reference population, for women aged 22–34 years, AMH levels <2.21 ng/mL were considered <25th percentile. For women aged 35–40 years, AMH levels <1.01 ng/mL were considered <25th percentile.

We used multivariable log-binomial regression to model the prevalence of AMH <1.0 ng/mL or AMH <25th percentile of comparison women by age, adjusting for age, BMI and hormonal contraception use in the past year. Model 1 adjusted for continuous age. Model two also adjusted for BMI (underweight: <18.5 kg/m^2^, normal weight: 18.5–<25 kg/m^2^, overweight: 25–<30 kg/m^2^ and obese: ≥30 kg/m^2^) and hormonal contraceptive use in the previous 12 months.

To determine the appropriate modelling specifications, we ran several models among the comparison women of AMH as a function of age, and plotted the predicted values of AMH against the actual values. We considered linear AMH and linear age, the log transformation of AMH with linear age, linear AMH with quadratic age and the log transformation of AMH with quadratic age. None of the other specifications appeared to be a better fit than linear AMH with linear age. We chose to not include a quadratic term for age in our models and to plot the predicted values of AMH treating it as a linear variable.

All statistical analyses were conducted using SAS V.9.4 (SAS Institute, Cary, North Carolina, USA).

## Results

Two women with SLE and five comparison women were excluded due to a diagnosis of PCOS. The final analytic sample included 83 comparison women without SLE, 68 women with SLE and no history of CYC treatment (SLE/CYC−) and 11 women with SLE with a history of CYC treatment (SLE/CYC+) (table 1). Among the comparison women, three women had AMH levels that were below the LOD of the Ultrasensitive assay and were therefore assigned a value of 0.054 ng/mL ($\frac{LOD}{\sqrt{2}})$). Among the women with SLE, seven had AMH levels below the LOD, all in the SLE/CYC− group and were assigned an AMH level of 0.054 ng/mL.

**Table 1:** Characteristics of study participants

Characteristic	SLE/CYC− (n=68)	SLE/CYC+ (n=11)	Comparison (n=83)
AMH in ng/mL (mean (SD))	2.99 (3.17)	1.17 (0.63)	3.26 (3.15)
Age at interview (n (%)), years			
22–30	25 (36.8)	6 (54.6)	6 (7.2)
31–34	9 (13.2)	2 (18.2)	20 (24.1)
35–37	15 (22.1)	3 (27.3)	29 (34.9)
38–40	19 (27.9)	0 (0.0)	28 (33.7)
Age at SLE diagnosis (n (%)), years			
18–20	17 (25.0)	3 (27.3)	
21–25	23 (33.8)	7 (63.6)	
26–30	14 (20.6)	1 (9.1)	
31–35	14 (20.6)	0 (0.0)	
Body mass index (n (%))			
Underweight (<18.5 kg/m^2^)	3 (4.4)	0 (0.0)	1 (1.2)
Normal weight (18.5–<25 kg/m^2^)	16 (23.5)	7 (63.6)	17 (20.5)
Overweight (25–<30 kg/m^2^)	19 (27.9)	2 (18.2)	31 (37.4)
Obese (≥30 kg/m^2^)	30 (44.1)	2 (18.2)	34 (41.0)
Hormonal contraception in previous 12 months (n (%))	14 (20.6)	2 (18.2)	24 (28.9)
Experienced menopause* (n (%))	3 (4.4)	0 (0.0)	0 (0.0)

*Amenorrhoea for 12 months or longer without resumption of menses.

AMH, anti-Müllerian hormone; CYC, cyclophosphamide.

The unadjusted means for the SLE/CYC− women (2.99 ng/mL, 95% CI: 2.22 to 3.76) and comparison women (3.26 ng/mL, 95% CI: 2.57 to 3.95) were similar, while for the SLE/CYC+ women, the mean AMH level was substantially lower (1.17 ng/mL, 95% CI: 0.75 to 1.59) (table 1). Women in the comparison group were slightly older than women with SLE with a median age of 36 (IQR: 33–38). SLE/CYC− women had a median age of 34.5 (IQR: 29–38) and SLE/CYC+ women had a median age of 30 (IQR: 28–35). SLE/CYC+ women were younger at the time of SLE diagnosis (median age: 22, IQR: 20–24) compared with SLE/CYC− women (median age: 24.5, IQR: 20.5–29). Over 40% of the SLE/CYC− women and the comparison women were obese. SLE/CYC+ women were less likely to be obese. Comparison women were more likely to have used hormonal contraception in the previous 12 months. Only three women in the entire analytic sample had experienced menopause at the time of interview, all in the SLE/CYC− group.

Women with SLE, both in age groups 22–34 and 35–40 years, were more likely to have AMH levels <1.0 ng/mL (table 2). Over 30% of SLE/CYC− women and over 50% of SLE/CYC+ women had AMH levels below 1.0 ng/mL, compared with 20.5% of comparison women. When examining the proportion of women with SLE who have AMH levels below the 25th percentile by age category of comparison women, the differences are more striking. By definition, 25% of comparison women have low AMH levels based on this metric, so the proportions were not included in the table. In contrast, 42.7% of SLE/CYC− women and over 70% of SLE/CYC+ women have AMH values below the 25th percentile of comparison women by age.

**Table 2:** Proportion of women with low AMH using different definitions

Age group (years)	AMH <1.0 ng/mL			AMH <25th percentile of comparison women		
	SLE/CYC− (n=68)	SLE/CYC+ (n=11)	Comparison (n=83)	SLE/CYC− (n=68)	SLE/CYC+ (n=11)	Comparison 25th percentile (ng/mL)
22–34	8 (23.5)	5 (62.5)	3 (11.5)	15 (44.1)	7 (87.5)	2.21
35–40	14 (41.2)	1 (33.3)	14 (24.6)	14 (41.2)	1 (33.3)	1.01
Total	22 (32.4)	6 (54.6)	17 (20.5)	29 (42.7)	8 (72.7)	

AMH, anti-Müllerian hormone; CYC, cyclophosphamide.

Controlling for age as a continuous variable, SLE/CYC− women were 1.5–1.6 times as likely to have AMH <1.0 ng/mL and AMH levels below the 25th percentile compared with women without SLE (table 3). These estimates changed only slightly after controlling for BMI and hormonal contraception use in the previous 12 months in addition to age. SLE/CYC+ women were nearly 3 times as likely to have AMH <1.0 ng/mL compared with comparison women (prevalence ratio (PR): 2.90, 95% CI: 1.29 to 6.51). When considering AMH below the 25th percentile as the outcome, the association was slightly attenuated but still prominent (PR: 2.43, 95% CI: 1.40 to 4.22). When comparing SLE/CYC+ women with SLE/CYC− women, SLE/CYC+ women were more likely to have AMH below 1.0 ng/mL (PR: 1.79, 95% CI: 0.81 to 3.95) and below the 25th percentile (PR: 1.57, 95% CI: 0.94 to 2.61).

**Table 3:** Adjusted prevalence ratios (PR) using different cut-points for low AMH values

	AMH <1.0 ng/mL		AMH below 25th percentile*	
	Model 1†	Model 2‡	Model 1†	Model 2‡
	PR (95% CI)	PR (95% CI)	PR (95% CI)	PR (95% CI)
SLE/CYC− vs comparison	1.68 (0.97 to 2.91)	1.62 (0.93 to 2.82)	1.55 (0.97 to 2.47)	1.55 (0.97 to 2.47)
SLE/CYC+ vs comparison	2.90 (1.45 to 5.78)	2.90 (1.29 to 6.51)	2.66 (1.59 to 4.45)	2.43 (1.40 to 4.22)
SLE/CYC+ vs SLE/CYC−	1.72 (0.91 to 3.24)	1.79 (0.81 to 3.95)	1.72 (1.09 to 2.70)	1.57 (0.94 to 2.61)

*25th percentile cut-point categories were assigned separately for those aged 22–34 and 35–40 years.

†Adjusting for age at interview (continuous).

‡Adjusting for age at interview (continuous), body mass index (underweight: <18.5 kg/m^2^, normal weight: 18.5–<25 kg/m^2^, overweight: 25–<30 kg/m^2^ and obese: ≥30 kg/m^2^) and hormonal contraception in the previous 12 months.

AMH, anti-Müllerian hormone; CYC, cyclophosphamide.

**Figure 1:**
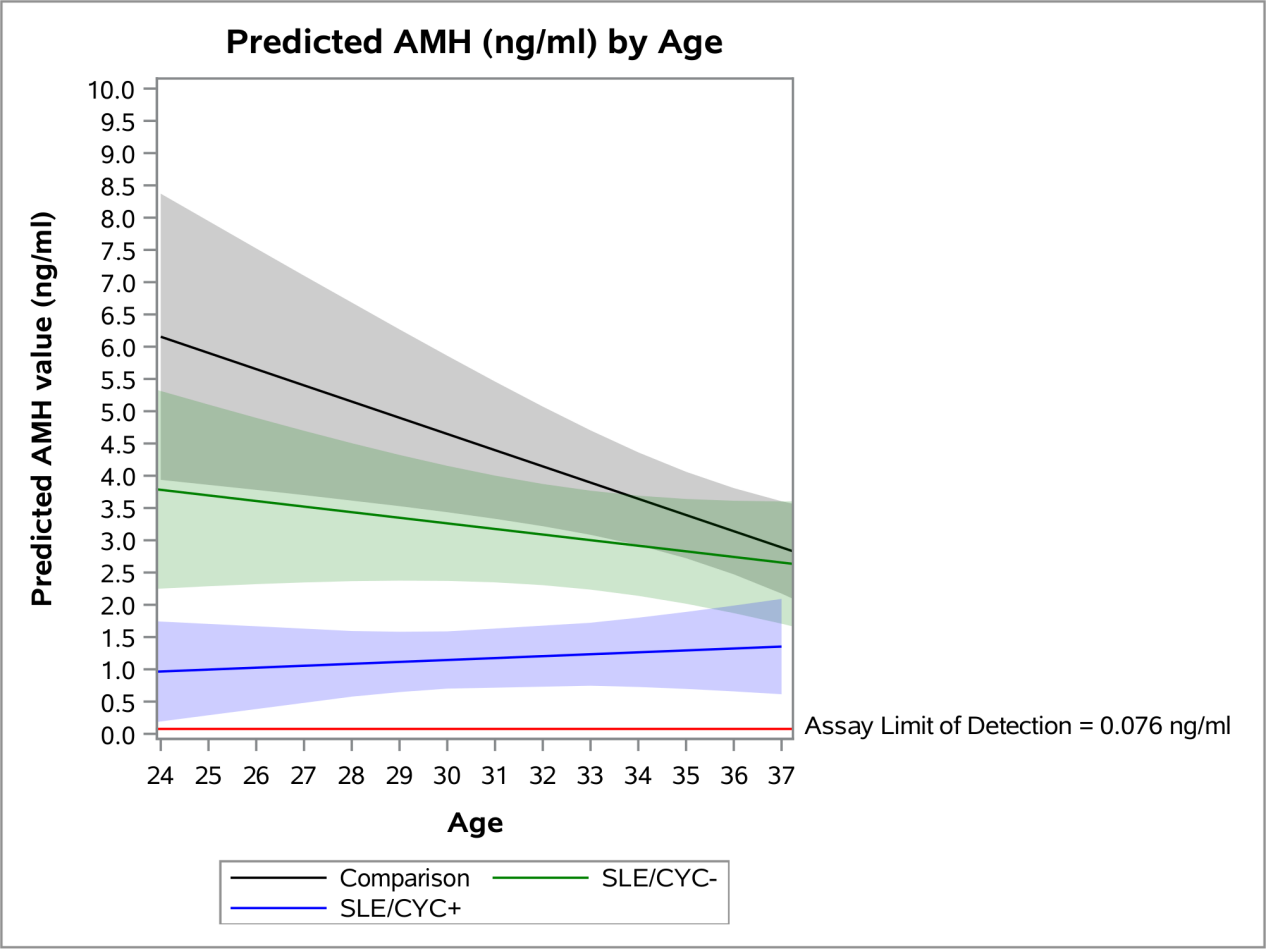
Predicted AMH values (ng/mL) by age with 95% CIs. AMH. anti-Müllerian hormone; CYC, cyclophosphamide.

Finally, we plotted the predicted AMH levels by age for each of the three exposure categories (figure 1). We restricted the age range of the plot to 24–37 due to the small number of observations of SLE/CYC+ women in the 22–23 and 38–40 age groups. At all ages, predicted AMH levels for the SLE/CYC+ group were noticeably lower than for the other two groups, and remained fairly flat with age. The SLE/CYC− group, while appearing to have lower AMH at younger ages, became more similar to the comparison group at older ages. However, it should be noted that the 95% CIs for the comparison group and the SLE/CYC− group overlap substantially.

## Discussion

Our analysis shows a slight reduction in AMH values after controlling for age, BMI and hormonal contraception use even among women with SLE who had never been treated with CYC. Women with SLE were 1.5–1.6 times as likely to have low values of AMH compared with women without SLE. Our plot of predicted values suggested that this difference was more pronounced at younger ages. This suggests an association between SLE and ovarian insufficiency. Diminished ovarian reserve may be caused by several different mechanisms among women with SLE, including autoimmune oophoritis, where autoantibodies target parts of the ovary which in turn causes ovarian inflammation and reduced follicle counts.^29^ While we were unable to examine disease damage in our study population, several studies have noted that high damage scores are associated with lower AMH levels.^10 30^ Our results are in contrast to a few studies that did not find an association between AMH and SLE without a history of CYC treatment.^10 17^ Our result is supported by a recent meta-analysis that did find an association between AMH and SLE among women not treated with CYC, although the authors noted that their pooled results were heavily influenced by one study that showed a very strong association.^31^

Previously, AMH was thought to be predictive of a woman’s ability to conceive. More recent studies, however, suggest that AMH is not associated with time to pregnancy in women attempting to conceive or necessarily lower in women who have reported infertility.^11 32 33^ However, AMH is valuable clinically as predictive of how well a woman will respond to fertility treatments. Assisted reproductive technologies are both considered safe in women with SLE with stable disease, and important to consider as women with SLE may be more likely to experience infertility, as shown in another paper by our group.^34 35^ Low AMH has also been identified as a predictor of time to menopause, including early menopause, defined as menopause before age 45 years.^7 8^ Time to menopause is important among women seeking or eventually seeking pregnancy as their fertile window may be shorter if menopause occurs early. In addition, time to menopause may also be a predictor of other health outcomes. It is generally shown that women with early menopause are also at an increased risk of cardiovascular disease.^36^ While the mechanism underlying the increased risk of cardiovascular disease after menopause remains controversial, low and/or declining levels of AMH have been shown to be associated with cardiovascular disease overall as well as coronary heart disease.^37 38^ Women with SLE who are diagnosed at younger ages or have been treated with CYC have been shown to be at risk of early menopause.^39^ As these women with SLE may be at greater risk of early menopause and women with SLE are already at greater risk of early cardiovascular disease than the general population, there is a need to examine how AMH, a potential predictor of early menopause, compares in women with SLE relative to the general population.^40 41^

Like other studies, we found that AMH is substantially lower among women with SLE who had been treated with CYC.^10–13^ While our sample of women treated with CYC was small, they were noticeably younger than both women with SLE who had not been treated with CYC and the comparison women, yet still had substantially lower AMH values. When predicted values of AMH were plotted by age, women treated with CYC appeared to have AMH values comparable to much older women not treated with CYC. This is also demonstrated where nearly all of the women treated with CYC younger than 35 years had AMH levels below the 25th percentile of the comparison group. It is well-established that CYC is a gonadotoxic agent that causes ovarian damage.^42^ At present, evidence of the effectiveness of gonadotropin-releasing hormone agonists (GnRH-a) for fertility preservation among women receiving CYC is not strong.^43 44^ It is also suggested that adolescents with SLE do not always receive fertility counselling prior to receiving gonadotoxic treatments.^45^ More work is needed to discern an effective counselling protocol for women with SLE receiving potentially gonadotoxic treatments.

Our analysis has several limitations that should be noted. First, we had a relatively small number of participants, especially when considering those exposed to CYC and those at the ends of the age distribution (<24 and >37 years). However, our study still represents the largest study of AMH among African-American women with SLE. Second, we did not have information on the cumulative dose or timing of CYC treatment, which has been shown to be negatively associated with AMH.^12^ We also did not have information on if women treated with CYC had also received GnRH-a therapy. In addition, women treated with CYC likely have more severe disease than women with SLE who were never treated with CYC. We cannot distinguish between the effect of disease severity and CYC among women treated with CYC, so there may be confounding by indication. However, the confounding effect of disease severity would likely need to be extremely strong to fully account for the association between CYC and AMH levels.^46^ By excluding women currently receiving kidney dialysis, we also have potentially excluded women with severe SLE. Finally, some research has suggested that individual trajectories of AMH over time are more salient predictors of important health outcomes than population-level averages by age.^38 47^ As this was a cross-sectional study, we were unable to examine individual trajectories of AMH over time.

This is the only study to have specifically examined AMH among African-American women with SLE. We were able to distinguish between women with SLE who had been treated with CYC and those that had not, and also included a comparison group of women without SLE. Our research suggests that even women with SLE not treated with CYC may have lower levels of AMH than women without SLE. This suggests that both the disease and treatment of SLE negatively impacts ovarian reserve and long-term ovarian function. Future research should examine trajectories of AMH in women with SLE with repeated measurements. In addition, the ability of AMH to predict other health outcomes, such as cardiovascular disease and the onset of early menopause among women with SLE, should be explored further.
